# The crystal structure of an antiparallel chair-type G-quadruplex formed by Bromo-substituted human telomeric DNA

**DOI:** 10.1093/nar/gkz221

**Published:** 2019-04-08

**Authors:** Yanyan Geng, Changdong Liu, Bo Zhou, Qixu Cai, Haitao Miao, Xiao Shi, Naining Xu, Yingying You, Chun Po Fung, Rahman Ud Din, Guang Zhu

**Affiliations:** 1 Division of Life Science, The Hong Kong University of Science and Technology, Clear Water Bay, Kowloon, Hong Kong SAR, China; 2 Institute for Advanced Study, The Hong Kong University of Science and Technology, Clear Water Bay, Kowloon, Hong Kong SAR, China; 3 State Key Laboratory of Molecular Neuroscience, The Hong Kong University of Science and Technology, Clear Water Bay, Kowloon, Hong Kong SAR, China

## Abstract

Human telomeric guanine-rich DNA, which could adopt different G-quadruplex structures, plays important roles in protecting the cell from recombination and degradation. Although many of these structures were determined, the chair-type G-quadruplex structure remains elusive. Here, we present a crystal structure of the G-quadruplex composed of the human telomeric sequence d[GGGTTAGG_8_GTTAGGGTTAGG_20_G] with two dG to 8Br-dG substitutions at positions 8 and 20 with *syn* conformation in the K^+^ solution. It forms a novel three-layer chair-type G-quadruplex with two linking trinucleotide loops. Particularly, T5 and T17 are coplanar with two water molecules stacking on the G-tetrad layer in a sandwich-like mode through a coordinating K^+^ ion and an A6•A18 base pair. While a twisted Hoogsteen A12•T10 base pair caps on the top of G-tetrad core. The three linking TTA loops are edgewise and each DNA strand has two antiparallel adjacent strands. Our findings contribute to a deeper understanding and highlight the unique roles of loop and water molecule in the folding of the G-quadruplex.

## INTRODUCTION

Telomeres are composed of repetitive DNA sequences located at the ends of chromosomes that function in maintaining chromosomal integrity and protection of chromosomal DNA from enzymatic end-degradation (1,2). The human telomere contains consecutive repeats of the DNA sequence d[GGGTTA] which can fold into four-stranded intramolecular structures known as G-quadruplexes (3). The G-quadruplex is a tetra-helical structure formed by guanine-rich sequences, consisting of stacked G-tetrad planes connected by Hoogsteen hydrogen bonds and stabilized by monovalent cations such as Na^+^ and K^+^ (4,5). It has been reported that the human telomeric G rich DNA sequence, d[GGGTTA]n, can fold into a variety of topologies including single or multi stranded G-quadruplex structures depending on the lengths, flanking nucleotides at the 5′ and 3′ ends and associated cations. Recently, G-quadruplexes have been revealed in human cells using an antibody and small molecules (6–8) and shown to play important roles in numerous processes such as DNA replication, recombination, transcription, translation and telomere maintenance (9–17). Therefore, these findings greatly boosted the attention toward G-quadruplexes as attractive therapeutic targets for anti-cancer drug design (8,18,19). In this light, the structural details are required to develop highly specific ligands capable of discriminating the diverse human telomeric G-quadruplex topologies/structures. Very recently, there has been a surge in the studies of the folding pathways and the relevant intermediates of G-quadruplex formation by using biophysical or molecular dynamics simulation approaches (20–31). All these studies unequivocally established the presence of intermediates during the folding pathway of G-quadruplex formation. However, some of the structural characterizations of these intermediates have remained elusive.

Currently, there are six distinct G-quadruplex topologies, eight structures, observed for human telomeric d[GGGTTA]n sequence including parallel, hybrid, antiparallel basket and (2+2) form (32–40) (Supplementary Figure S1). All of these reported structures contain the central d[(GGGTTA)_3_GGG] core, named *htel21*, and the final folding depends on the flanking sequences at the 5′ and 3′ ends. Nonetheless, accumulating experimental evidence and molecular dynamics simulation results point to the existence of unreported antiparallel chair-type G-quadruplex structures (24,25,29,31). Especially, an antiparallel chair-type G-quadruplex, a fold previously unknown to human telomeric repeats, has been reported to be formed by a human telomeric variant *htel21*T_18_ with a T substitution at A18 of *htel21* further corroborating the possible existence of the similar antiparallel chair-type fold adopted by *htel21* (41). However, the failure of crystallization of *htel21* indicated the structural polymorphism possibly making the structural elucidation difficult. Interestingly, through NMR examination we found that the guanines at positions 8, 20 in wild-type *htel21* adopt *syn*. It is well known that the conformational heterogeneity could be greatly resolved by substituting core *syn*-guanine(s) with 8-bromo-guanines (8Br-dG) that favour the *syn* conformation (34,38,42–44). Consequently, double dGs to 8Br-dG substitutions at positions 8, 20 in *htel21* allowed us to obtain a crystal with the good quality of diffraction data. Meanwhile, we have shown that the double 8Br-dG substituted *htel21* has the same *syn* conformations at G8 and G20 as that of *htel21* in K^+^ solution based on the NMR study.

Here, we presented a novel crystal structure of a three-layer chair-type G-quadruplex using 8Br-dG substitutions at 8 and 20 positions of *htel21*, termed as *htel21*_Br-8,20. It adopts a unique chair-type G-quadruplex fold with three G-tetrad layers. Particularly, T5 and T17 are coplanar with two water molecules stacking on the G-tetrad layer in a sandwich-like mode through a coordinating K^+^ ion and an A6•A18 base pair. While a twisted Hoogsteen A12•T10 base pair caps on the G-tetrad core. This antiparallel chair-type G-quadruplex is characterized by a 5′-*syn*G•*anti*G•*anti*G–*syn*G•*syn*G•*anti*G–*syn*G•*anti*G•*anti*G–*syn*G•*syn*G•*anti*G-3′ strand arrangement with edgewise-edgewise-edgewise loops from the 5′ to 3′ direction. Our result emphasizes the significance of loop and water molecules in the G-quadruplex folding. To our knowledge, herein for the first time, we reported the high resolution structure of an antiparallel chair-type G-quadruplex formed by human telomeric sequence. Additionally, the structure provides the atomic details of the chair-type G-quadruplex for the computer-aided ligand screening studies.

## MATERIALS AND METHODS

### Sample preparation

The single DNA strands including unlabelled, site-specific 5% ^15^N,^13^C-labeled low-enrichment and 8Br-dG substituted nucleotides were purchased from Integrated DNA Technologies (IDT) and Takara. DNA sample at 0.1 mM (single strands) was annealed by heating to 95°C for 15 min, followed by slow cooling to room temperature overnight in an annealing buffer containing 70 mM KCl and 20 mM potassium phosphate (pH 7.0). For NMR examination, the DNAs buffer was added with 10% D_2_O. For crystallization, the DNAs were purified by FPLC using the mono Q column (GE healthcare) and buffer exchanged into 20 mM Tris, 100 mM KCl at pH 7.0.

### NMR spectroscopy

Nuclear magnetic resonance (NMR) experiments were performed on INOVA 500 MHz and 800 MHz Varian spectrometers at 25°C. Assignment of guanine imino protons in 5% ^15^N,^13^C site-specifically labeled samples was achieved by performing 1D ^15^N-filtered HSQC experiments. Guanine H8 protons were identified by performing 2D HMBC experiment at natural abundance or 2D ^13^C-HSQC experiments on 5% ^15^N,^13^C site-specifically labeled DNA samples. The 2D NOESY spectra were collected at 25°C. Spectra were processed with the program nmrPipe (45,46) and analyzed by using the software Sparky (http://www.cgl.ucsf.edu/home/sparky/).

### Circular dichroism Spectroscopy

Circular dichroism (CD) spectra were recorded at 25°C on an Applied Photophysics Chirascan CD spectrometer using 1 mm path length quartz cuvette with sample volume 400 μl. The DNA oligonucleotides were prepared in 20 mM potassium phosphate buffer (pH 7.0) containing 70 mM KCl at concentration of 15 μM (single strands). The melting curves were obtained by monitoring at 290 nm and temperature ranged from 25°C to 95°C with a heating rate at 1°C/min. Data were processed by the Boltzmann sigmoid equation (Prism).

### Crystallization

The DNA sample at concentration of 1.8 mM was initially screened using Natrix HT kit (Hampton Research) with the sitting-drop vapor–diffusion technique at 16°C. Small crystals were grown from buffer containing 12 mM NaCl, 80 mM KCl, 40 mM Sodium Cacodylate trihydrate pH 6.0, 50% MPD, 12 mM spermine tetrahydrochloride within two weeks. Then the condition was optimized two-dimensionally with different pH versus concentration of MPD using hanging-drop vapor–diffusion method. The drop ratio of sample to reagent was also varied and large crystals were obtained with a 4:1 drop ratio of 0.8 μl DNA plus 0.2 μl reagent within one month.

### Data collection and structure determination

For data collection, the crystal was directly flash-cooled in liquid nitrogen. The diffraction data set was collected on beamline BL19U at Shanghai Synchrotron Radiation Facility (SSRF) using a PILATUS3 6M detector with the peak wavelength of Br (λ = 0.91904 Å). Intensity data were integrated and scaled by HKL3000 package (47). The structure was determined by single wavelength anomalous dispersion (SAD) method. The positions of bromine atoms were found and refined, then the phases were calculated using phenix.autosol (48). The experimental SAD electron density map was of excellent quality and model tracing was completed manually in Coot (49). The structure was refined with phenix.refine (48). The final refinement statistics were summarized in Supplementary Table S1. All figures of G-quadruplex structure were prepared using PyMOL (http://www.pymol.org).

## RESULTS

### Guanines at positions 8, 20 in *htel21* adopt *syn* conformation

The CD spectra of *htel21* displayed two positive peaks at ∼250 and ∼290 nm (Supplementary Figure S2) signifying that it predominantly adopts antiparallel G-quadruplexes in the presence of K^+^ ion. The shoulder at ∼270 nm indicated the existence of multiple conformations such as the hybrid (3+1) G-quadruplexes. However, the CD spectroscopy cannot provide detailed structural arrangement to distinguish other antiparallel conformations from the known antiparallel basket G-quadruplex. It has been reported that substitution of guanosines in the *syn* conformation with 8Br-dG could overcome the conformational heterogeneity and increase the thermal stability of G-quadruplexes (34,38,42–44). Since G1, G7, G8, G13, G19 and G20 have *syn* conformations in the *htel21*T_18_, a human telomeric variant d[(GGGTTA)_2_GGGTTTGGG] with a T substitution at A18 of *htel21* forming an antiparallel chair-type G-quadruplex (41), initially we carried out several G-to-8Br-dG substitutions of these guanines in the *htel21* (Supplementary Figure S2). As shown in the Supplementary Figures S2B and C, we found that a double 8BrG substitution at G8 and G20 for the *htel21* had significantly improved the quality of the spectrum. The positive bands around ∼290 and ∼250 nm shown in the CD spectra of *htel21* and the double 8Br-dG substitutions at G8 and G20 for the *htel21* indicated that they should adopt the same G-quadruplex in K^+^ solution, although there is a negative band near 260 nm in the CD spectrum of the double 8Br-dG substituted *htel21* instead of a small positive shoulder for *hte21*.

**Figure 1.**
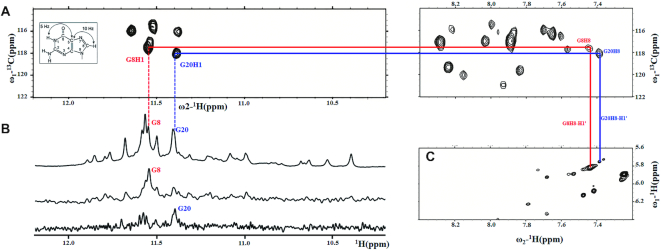
(**A**) The imino and H8 regions of the 2D HMBC spectrum showing correlation between imino proton and H8 proton within guanosine bases of *htel21* sequence. Inset: through-bond correlations between guanosine imino and H8 protons via ^13^C (at 5-position) at natural abundance using long-range J couplings. 2D HMBC spectrum was recorded at 800 MHz, 25°C in 10% D_2_O, 70 mM KCl, 20 mM potassium phosphate buffer (pH 7.0). (**B**) The imino region of 1D ^1^H-NMR spectra of *htel21* with the assignment of guanine bases at positions 8 and 20 indicated over the reference spectrum on the top. Guanine imino protons at positions 8 and 20 were assigned with 1D ^15^N-filtered HSQC spectra of samples containing site-specific low-enrichment (5%) ^15^N-labelled oligonucleotides at the indicated positions. Spectra were recorded at 800 MHz (1D ^1^H-NMR spectrum and 1D ^15^N filtered HSQC spectra) at 25°C in 10% D_2_O, 70 mM KCl, 20 mM potassium phosphate buffer (pH 7.0). (**C**) Expanded ^1^H-^1^H NOESY spectrum (50 ms mixing time) correlating base H8 and sugar H1′ protons of *htel21*. The intraresidue guanosine H8-H1′ cross-peaks indicate *syn* glycosidic bonds.

Consequently, to further investigate the conformation of guanines at the positions 8 and 20 in *htel21*, we unambiguously assign the imino and H8 protons of G8 and G20 by 1D ^15^N-filtered HSQC spectra using low-enrichment (5%) ^15^N site-specific labelled oligonucleotides and 2D HMBC experiment (Figure 1A and B). Based on the unambiguous assignments of the H8 protons, two separated intraresidue H8-H1′ peaks can be clearly identified in the 2D NOESY spectrum acquired at 50 ms mixing time indicating the *syn* conformations of G8 and G20 in *htel21* (Figure 1C).

### An antiparallel G-quadruplex adopted by *htel21*_Br-8,20

As the NMR data demonstrated that G8 and G20 could adopt *syn* conformation in *htel21*, we carried out structural study on two *htel21* samples termed as *htel21*_Br-8 and *htel21*_Br-20 corresponding to 8Br-dG substitutions at position 8 and 20, respectively (Supplementary Figure S3). The CD spectra of *htel21*_Br-8 and *htel21*_Br-20 showed one positive peak at 290 nm, typical for antiparallel G-quadruplexes (Supplementary Figure S2) (50). The CD melting experiments showed an increase of *T*_m_ by ∼3–4°C for the *htel21*_Br-8 and *htel21*_Br-20 indicating higher thermal stability compared to the wild-type *htel21* (Supplementary Figure S3).

However, these two samples could not be crystalized successfully for X-ray crystallographic study. To obtain X-ray diffractive crystals, a double 8Br-dG substitution at 8 and 20 positions in *htel21*, named as *htel21*_Br-8,20, was designated for crystallization. To confirm that the *syn* conformation adopted by G8 and G20 simultaneously in one *htel21* DNA molecule, we further investigated the conformations of G20 in *htel21*_Br-8 and G8 in *htel21*_Br-20 with ^15^N,^13^C site-specific labelled (5% isotope enrichment) oligonucleotides and recorded 2D ^13^C-HSQC spectra. The H8 proton resonances of G20 in *htel21*_Br-8 and G8 in *htel21*_Br-20 were assigned subsequently (Supplementary Figures S4). Interestingly, the intraresidue H8-H1′ peaks in the 2D NOESY spectrum acquired at 50 ms mixing time clearly indicated the *syn* conformation of G20 in *htel21*_Br-8 and G8 in *htel21*_Br-20 respectively (Supplementary Figures S4). Strikingly, the CD spectrum of *htel*_Br-8,20 displayed a similar profile with *htel21* except the disappearance of the shoulder ∼270nm indicating the formation of a predominant antiparallel G-quadruplexes (Supplementary Figure S3). An increase of *T*_m_ by ∼3°C in the *htel21*_Br-8,20 also indicated a higher thermal stability compared to the *htel21* (Supplementary Figure S3). These results indicated that the double 8Br_dG substitution did not introduce artifacts in the case presented here. Finally, the X-ray diffractive crystals of *htel21*_Br-8,20 were successfully obtained.

### Overall crystal structure of *htel21*_Br-8,20

**Figure 2.**
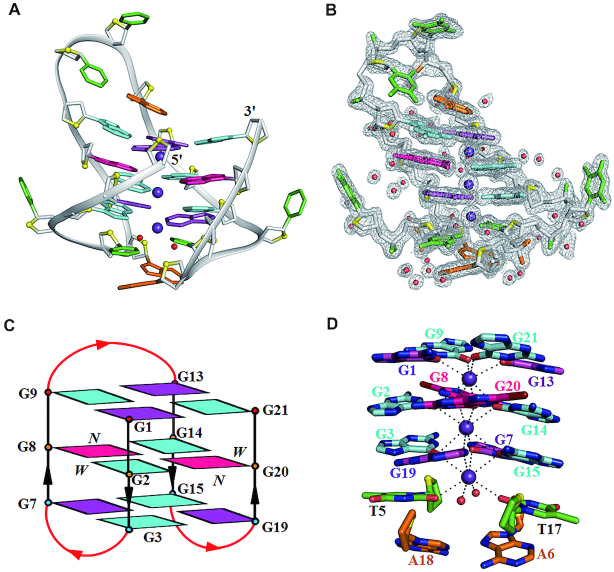
Crystal structure of *htel21*_Br-8,20. (**A**) Ribbon view of the crystal structure of *htel21*_Br-8,20. (**B**) The electron density corresponds to the final 2Fo-Fc map contoured at 1.0 σ. *anti* and *syn* guanine bases are colored in cyan and magenta, respectively; 8Br-dG bases are in hotpink; thymine bases are in green; adenine bases are in orange; phosphate backbone is in gray; water oxygens are in red; and O4′ oxygens are in yellow. Potassium ions and water molecules are colored in purple and red, respectively. (**C**) Schematic structure of *htel21*_Br-8,20. Arrows represent the 5′-to-3′ strand progression. The backbones of the core and loops are colored in black and red, respectively. W and N represent wide and narrow grooves. (**D**) Expanded view of the K^+^ ion in the G-quadruplex of *htel21*_Br-8,20. The coordination interactions between K^+^ ion and oxygen atom are shown as dashed lines. The *htel21*_Br-8,20 is shown in sticks, water molecules as red spheres, and potassium ions as purple spheres.

**Figure 3.**
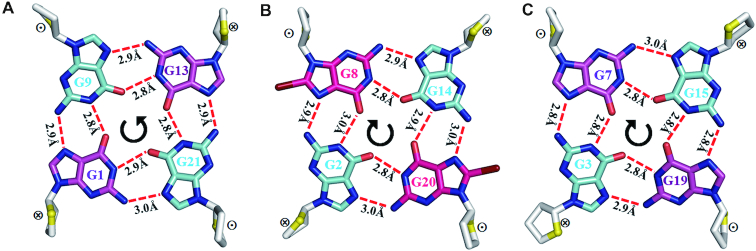
Conformations of the G1•G9•G13•G21, G2•G8•G14•G20 and G3•G7•G15•G19 layers. The hydrogen bonds are shown as dashed red lines; ⊗: 5′→3′ sugar direction is pointing into the paper; ⊙: 5′→3′ sugar direction is pointing toward viewers; *anti* and *syn* guanine bases are colored in cyan and magenta, respectively; 8-Bromosubstituted guanine bases are in hotpink.

**Figure 4.**
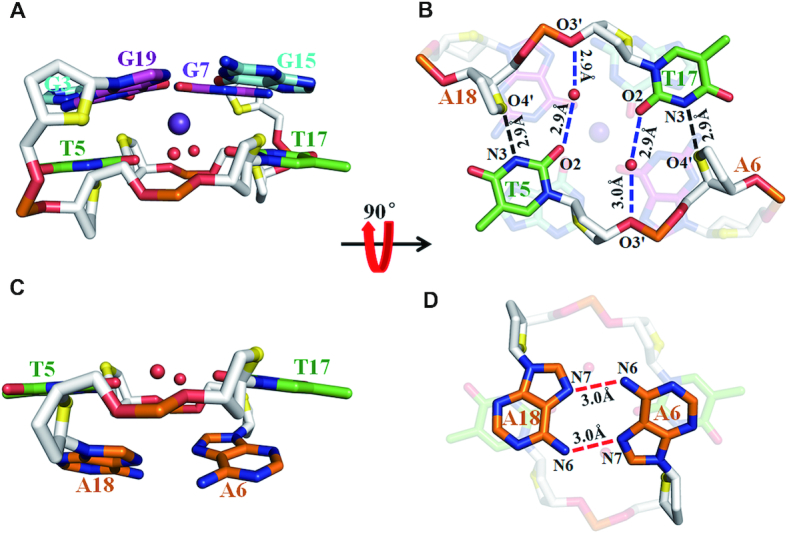
The structures of loops T4-T5-A6 and T16-T17-T18 in *htel21*_Br-8,20. (**A**) The T5 and T17 bases are coplanar with two water molecules stacking with a G-tetrad layer, G3•G7•G15•G19, by a coordinating K^+^ ion. (**B**) The T5 and T17 are stabilized by the hydrogen bonds between the thymine and sugar rings of adenine, T5N3-A18O4′ and T17N3-A6O4′; two water molecules bridges between the O2 atoms of T5, T17 and the O3′ atoms of T17, T5. (**C** and **D**) The A6 and A18 residues form an A6•A18 base pair through N7-N6 hydrogen bonds capping on the T5 andT17. The hydrogen bonds are shown in dashed lines. Phosphate backbone is in gray; phosphorus and phosphates oxygens are orange and red; water oxygens are in red; and O4′ oxygens are in yellow. Potassium ions and water molecules are colored in purple and red, respectively.

The crystal structure of *htel21*_Br-8,20 was solved by Br-SAD method to ∼1.4 Å resolution in the space group of P6_5_22 (Figure 2A and Supplementary Table S1). The electron density was well defined for all of the nucleotides (Figure 2B and Supplementary Figure S5). There is only one G-quadruplex molecule in an asymmetric unit. The crystal structure shows an intramolecular antiparallel-stranded right-handed chair-type G-quadruplex, comprising three G-tetrad layers in which the hydrogen-bond directionalities of the three G-tetrads are anti-clockwise, clockwise and clockwise (G1←G9←G13←G21, G2→G8→G14→G20 and G3→G7→G15→G19) with all Hoogsteen N1-O6 and N2-N7 hydrogen bonds intact (Figures 2A, C and 3). The glycosidic conformations of guanines around the tetrads are *syn*•*anti*•*syn*•*anti*. The G-tetrad core are connected by three edgewise side TTA loops. There are two wide and two narrow grooves. The first and third loops (at the bottom) span narrow grooves, while the second loop (on the top) spans a wide groove (Figure 2C). There are three equal-spaced potassium ions, featuring an antiprismatic coordination environment, lying along the axis within the central core of the quadruplex with distance of ∼3.5 Å between K ions (Figure 2D). Two of the potassium ions are sandwiched between G-tetrads with an average distance of 2.8 Å from the O6 carbonyl atoms of the guanine residues, while another potassium ion is located between the G3•G7•G15•G19 G-tetrad and T5, T17 with an average distance of 2.9 Å from the O6 carbonyl atoms of the guanine residues and 2.8 Å from the O2 atoms of T5, T17 and the additional two water molecules respectively (Figures 2D and 4A).

**Figure 5.**
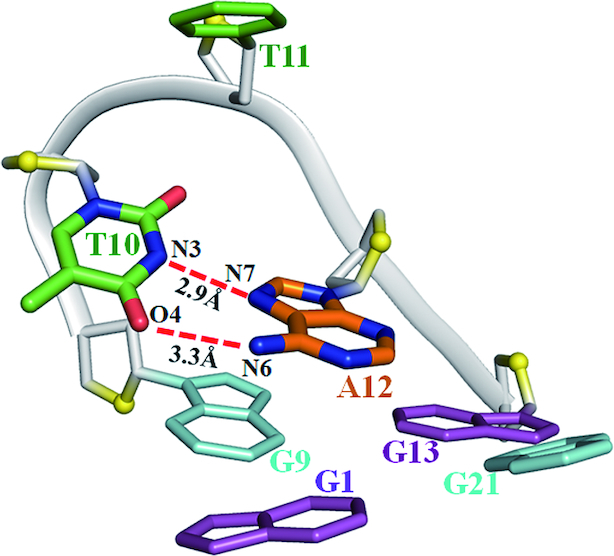
The structures of loops T10-T11-A12 in *htel21*_Br-8,20. A twisted Hoogsteen A12•T10 base pair through T10N3-A12N7 and T10O4-A1N6 hydrogen bonds stacks with G1•G9•G13•G21 layer. The T11 residue is almost parallel with and far away from the G1•G9•G13•G21 layer. The hydrogen bonds are shown in dashed lines. Phosphate backbone is in gray; and O4′ oxygens are in yellow.

Strikingly, the T5 and T17 residues form the hydrogen bonds between pyrimidine rings of thymine and sugar rings of neighboring adenine, T5N3-A18O4′ and T17N3-A6O4′ (Figure 4A and B). The T5 and T17 residues are coplanar with two water molecules stacking on the G-tetrad layer, G3•G7•G15•G19, through a coordinating K^+^ ion (Figures 2D, Figure 4A and B). Especially, as shown in the Figure 4B, two water molecules bridges between the O2 atoms of T5 and T17 and the O3′ atoms of T17 and T5, maintaining the coplanar conformation of T5 and T17. The A6 and A18 nucleotides form an A6•A18 base pair through N7-N6 hydrogen bonds which caps on coplanar T5, T17 residues (Figure 4C and D). The G-tetrad layer, G3•G7•G15•G19, T5, T17 and an A6•A18 base pair form a sandwich-like stacking mode which should highly stabilize the chair-type G-quadruplex. The T4 and T16 residues bulge out from the quadruplex core and interact with T16′ and T4′ from neighboring molecules through π-π interaction, facilitating crystal packing (Supplementary Figure S6). The loop containing T10, T11 and A12 locates the exterior of the quadruplex core. The A12 stacks and parallels with the G-tetrad layer, G1•G9•G13•G21. The temperature factor of T10, ∼69.6 Å^2^, is extremely higher than the average temperature factor of the overall crystal structure, 31.0 Å^2^ (Supplementary Table S1), indicating the high flexibility of T10. However, it forms a twisted Hoogteen A12•T10 base pair through T10N3-A12N7 and T10O4-A12N6 hydrogen bonds capping on the G1•G9•G13•G21 layer, which partially stabilizes T10 nucleotide (Figure 5). The T11 nucleotides are almost parallel and far away from the G1•G9•G13•G21 layer. Interestingly, there is a crystal packing interaction for A12 and T11′ of neighboring molecule, establishing a canonical Watson-Crick A12•T11′ base pair stacking with the G1•G9•G13•G21 layer. There is also a ‘tail to tail’ crystal packing interaction mediated by the π–π interactions from A18-A18′ and A6-A6′. (The prime (′) notation signifies that the two bases belong to separate oligonucleotide strands) (Supplementary Figure S6).

We analyzed the distribution of backbone dihedral angles (α, β, γ, δ, ϵ and ζ describe DNA backbone), glycosidic torsion angles (χ) and sugar puckers of three TTA loops and summarized the data in Supplementary Table S2 and the Supplementary Figure S7. The observed values indicate that the conformations of the T4–T5–A6 and T16–T17–T18 loops are almost same but different from the conformation of T10–T11–A12. Comparison with the loop conformations of the eight known human telomeric structures shown in the Supplementary Figure S1, interestingly, the third edgewise loop in the antiparallel (2+2) form observed for d[(TTAGGGTTA)_4_TTA] G-quadruplex in Na^+^ solution and the second edgewise loop in the (3+1) Form 2 observed for d[TA(GGGTTA)_3_GGGTT] display the close conformation with the of the T4–T5–A6 and T10–T11–A12 respectively.

## DISCUSSION

It is speculated that the human telomeric DNA sequence can adopt an antiparallel chair-type G-quadruplex fold in K^+^ solution (43,51). The presence of this fold in multiple folding pathways of human telomeric G-quadruplexes is also supported by previous FRET and MD studies (24,25,31). However, its high-resolution structure has remained unresolved. Recently, an antiparallel chair-type G-quadruplex structure formed by a human telomeric variant d[(GGGTTA)2GGGTTTGGG] termed as *htel21*T_18_ with a T substitution at A18 of *htel21* was solved by NMR (41). The structure of *htel21*T_18_ further corroborated the possible existence of the similar antiparallel chair-type fold adopted by *htel21*. However, the conformational heterogeneity of *htel21* makes structural elucidation difficult. The conventional methods to overcome the conformational heterogeneity for structural studies on G-quadruplexes are judiciously manipulating flanking sequences and/or substituting base-analogues (52). In our case here, the method of base-analogue substitution on guanines was chosen to overcome the conformational heterogeneity and the shortest sequence of *htel21* was retained. Especially, it is well recognized that the conformational heterogeneity could be greatly resolved by substituting core *syn*-guanine(s) with 8Br-dG(s) that favour the *syn* conformation (34,38,42–44). Therefore, we had to determine the conformations of guanines in *htel21* before substituting guanine with 8Br-dG. Fortunately, several G-to-8Br-G substitutions in the *htel21* based on the structure of *htel21*T_18_ provided the vital clues for the possible conformations of guanines in *htel21* (Supplementary Figure S2C). Subsequently, our NMR data demonstrated that the G8 and/or G20 could adopt *syn* glycosidic torsion angles (Figure 1). The further NMR investigation showed the *syn* conformations of G20 in *htel21*_Br-8 and G8 in *htel21*_Br-20, respectively. This result confirmed that both G8 and G20 can adopt *syn* conformations simultaneously in a single G-quadruplex among other possible structures of the wild-type *htel21* as indicated by NMR study (Supplementary Figure S3), Finally and importantly, this double dG substituted DNA allowed us to obtain a crystal of sufficient quality amenable for structural study.

**Figure 6.**
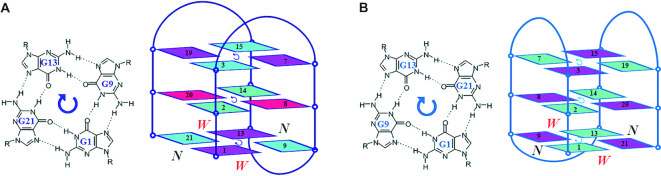
Comparison of the structural features of *htel21*_Br-8,20 (blue) and *htel21*T_18_ (light blue). (**A**) G1•G21•G13•G9 quartet and topology of *htel21*_Br-8,20. (**B**) G1•G9•G13•G21 quartet and topology of *htel21*T_18_. N and W represent the narrow and wide groove.

The crystal structure of *htel21*_Br-8, 20 adopts an intramolecular antiparallel chair-type G-quadruplex, comprising three G-tetrad layers connected by three edgewise side TTA loops. Strikingly, the T5 and T17 residues are coplanar with two water molecules stacking on the G-tetrad layer, G3•G7•G15•G19, through a coordinating K^+^ ion (Figures 2 and 4). The G-tetrad layer, G3•G7•G15•G19, an A6•A18 base pair and coplanar T5, T17 residues form a sandwich-like stacking mode and the stacking interaction between them play an important role in stabilizing the chair-type G-quadruplex fold and driving conformational equilibrium toward the three-layer chair-type fold. These special features cannot easily be extracted from NMR structural study of G-quadruplex. The three G-tetrads display the out-of-plane deviation, defined as the distance between the centers of mass of the inner tetragon formed by O6 atoms and outer tetragon formed by N9 atoms in G-tetrad (53), of 0.5, 0.04 and 0.04 Å for G1•G9•G13•G21, G2•G8•G14•G20 and G3•G7•G15•G19 respectively. The strongest deviation from planarity of G1•G9•G13•G21 is possibly caused by the formation of the Hoogsteen A12•T10 base pair and the π–π stacking interaction between the Hoogsteen A12•T10 base pair with the G1•G9•G13•G21 layer (Supplementary Figure S6). Although the central G-tetrad G2•G8•G14•G20 displays a small deviation from planarity, a propeller twist is observed for the 8Br-dG substituted G8 and G20 possibly caused by the large radius of bromine atom (Figures 2A and 3). Comparing with the NMR structure of *htel21*T_18_, which is a compact antiparallel G-quadruplex with three G-quartets and three lateral loops, where the A6•T18 base pair is observed across the lateral loops capping on the G-tetrad core at the same side (41),the high-resolution structure of *htel21*_Br-8,20 determined here uncovers a similar antiparallel type topology with three G-quartets and three lateral loops. When these two G-quadruplexes are placed in a common reference frame developed by Webba da Silva and co-workers (54,55) as shown in Figure 6, the strands and lateral (l) loops in *htel21*_Br-8,20 are designated as anticlockwise (–) and overall topology is (−l,−l,−l). While in *htel21*T_18_, the three lateral loops are designated as clockwise which corresponds to (+l,+l,+l) type topology. Overall, *htel21*T_18_ and *htel21*_Br-8,20 are similar in terms of the bases which comprise the individual G-tetrad layers, directionality of hydrogen bonds within each G-tetrad layer and glycosidic conformation of guanine residues along the sequence and in the G-tetrad core. However, *htel21*T18 and *htel21*_Br-8,20 differ in the donor-acceptor directionalities in each individual G-tetrad layer. For example, in the *syn*G1–*anti*G21 base pair, the donor of hydrogen bond in *htel21*_Br-8,20 is G1, whereas it is G21 in *htel21*T_18_. This reversal of hydrogen bond directionality results in the reversal of width of the grooves such as the *syn*G1→*anti*G21 is in a narrow groove in *htel21*_Br-8,20, whereas *anti*G21→*syn*G1 in *htel21*T18 is in a wide groove. Consequently, the first and the third lateral loops in *htel21*_Br-8,20 span wide grooves, whereas the middle loop spans a narrow groove. However, the first and third lateral loops in *htel21*T_18_ span narrow grooves and the middle loop spans a wide groove. In considering the conformational heterogeneity of *htel21* indicated by CD and NMR data, the G-quadruplex formed by *htel21*T_18_ could also be potentially adopted by *htel2l* in K^+^ solution.

Recently, two similar cases in which two distinct G-quadruplexes with the similar topology are observed for the same oligonucleotide have been reported. One is the hexanucleotide GGGGCC (G4C2) repeat linked with the most common genetic cause of amyotrophic lateral sclerosis (ALS) and frontotemporal dementia (FTD) which can form (+l,+l,+l) and (−l,−l,−l) type antiparallel chair-type G-quadurplexes with four G-tetrad layers (56). While, the other is a four-repeat human telomere DNA sequence d[TAGGG(TTAGGG)_2_TTAGG], named as TA_*htel21*_ΔG, which can form (+l,d,−l) and (−l,d,+l) type antiparallel basket-type G-quadruplexes with two G-tetrad layers (57). Interestingly, the two distinct G-quadurplexes in each case formed by ALS/FTD-G4C2 and TA_*htel21*_ΔG are reversible by tuning pH value. Importantly, the water•T5•water•T17 pseudo tetrad observed in our crystal structure of *htel21*_Br-8,20 provides a glimpse into understanding the mechanisms of the pH-dependent conformational switch of G-quadruplex. The change of pH value may affect the localization and residence time of water molecules in the water•T5•water•T17 pseudo tetrad and consequently leads to the conformational reversal.

To date, three types of antiparallel unimolecular human telomeric DNA G-quadruplexes with known structures have been reported, including the antiparallel basket type with three G-tetrad layers formed by 22-mer d[A(GGGTTA)_3_GGG] in Na^+^ solution, named as A_*htel21* (Supplementary Figure S1A) (33); the antiparallel basket type G-quadruplex with two G-tetrad layers adopted by the d[(GGGTTA)_3_GGGT] in K^+^ solution, shorted for *htel21*_T (Supplementary Figure S1E) (34), and the antiparallel G-quadruplex with (2+2) topology in Na^+^ solution observed by d[TTA(GGGTTA)_3_GGGTTA], called TTA_*htel21*_TTA (38) (Supplementary Figure S1F). They differ from the structure of *htel21*_Br-8,20 in the strand orientations, loops, the *syn/anti* arrangements and grooves in the G-tetrad core in the following four aspects. First, in A_*htel21, htel21*_T and TTA_*htel21*_TTA structures, two adjacent strands point up and two remaining strands point down (up–up–down–down), whereas each G-tetrad in *htel21*_Br-8,20 is oriented in the opposite direction with respect to its two neighboring ones (up–down–up–down). Second, A_*htel21* and *htel21*_T contain two edgewise loops and one diagonal loop, and TTA_*htel21*_TTA has two edgewise loops and one propeller loop. While in *htel21*_Br-8,20, all the loops are edgewise. Third, the *syn/anti* arrangement in each G-tetrad layer of A_*htel21, htel21*_T and TTA_*htel21*_TTA is *syn*·*syn*·*anti*·*anti*, whereas that in *htel21*_Br-8,20 is alternative (*syn*·*anti*·*syn*·*anti*). Finally, the chair-type *htel21*_Br-8,20 contains two wide and two narrow grooves, which are different from the other three structures with one wide groove, one narrow groove and two medium grooves commonly observed for the (3+1) form. These novel features of *htel21*_Br-8,20 were not observed previously in the context of human telomeric DNA repeats. Additionally, *htel21*_T consists two additional T•T base pairs and base triples in the loops capping on both ends of the two G-tetrad core showing the higher stability with melting temperature *T*_m_ ∼ 62.8 (34). Consequently, the higher thermal stability of *htel21*_Br-8,20 with *T*_m_ ∼77 might be explained by the observation of the sandwich-like stacking mode comprising the A6•A18 base pair, the coplanar water-mediated T5, T17 residues and the G-tetrad layer.

The structure of *htel21*_Br-8,20 is also distinct from known chair-type G-quadruplex structures formed by non-human telomeric sequences. Thrombin aptamer d[GGTTGGTGTGGTTGG] forms a unimolecular chair-type G-quadruplex solved by NMR consisting of two G-quartets connected by two TT loops forming a potential T•T base pair and one TGT loop (53,58). A sequence variant of human telomeric sequence d[AGGG(CTAGGG)_3_] adopts a two G-tetrad layer G-quadruplex involving two G-tetrads sandwiched between a G•C base pair and a G•C•G•C tetrad layer (59). A chair-type topology adopted by a *Bombyx mori* telomeric sequence d[TAGG(TTAGG)_3_] consists of a two-layer antiparallel G-tetrad core and three edgewise loops (60). Interestingly, the sequence, d[GGCGAGGAGGGGCGTGGCCGGC], in the promoter of *c-KIT* proto-oncogene can also form a chair-type antiparallel G-quadruplex with two G-tetrad layers and an additional G•C base pair in the loops (61). Recently, it was reported that an hexanucleotide GGGGCC (G4C2) repeat of the *C9orf72* gene in human, which is the most common genetic cause of amyotrophic lateral sclerosis (ALS) and frontotemporal dementia (FTD), adopts an antiparallel four-layer chair-type G-quadruplex with or without C•C/C+•C base pair in the three lateral CC loops (62–64). Whereas the chair-type G-quadruplex of *htel21*_Br-8,20 contains three layers of G-tetrad and the various conformation such as the A•A base pair in the loops.

Recent thermodynamics and kinetics studies in the unfolding/folding pathway of the human telomeric G-quadruplex formation revealed the great structural diversity for the same sequence. This inherent structural diversity of G-quadruplex hinders the rational design of drugs that bind specifically to G-quadruplexes due to the lack of high-resolution structural details, which cannot be provided by the low-resolution spectroscopy such as CD and FRET. In the previous study, the single molecule FRET experiment showed that the formation of the chair-type G-quadruplex is more favourable than the 2-tetrad basket and hybrid-2 conformations during the folding process, indicating that the chair-type G-quadruplex may have a higher population compared to other types of telomeric G-quadruplexes. Significantly, our atomic resolution structure contributes to a deeper understanding of the determinants during the folding pathway of G-quadruplex formation.

Experimental studies indicate that the long human telomeric sequences form intramolecular structures involving consecutive blocks of single G-quadruplex structures. To date, two main models have been proposed: the beads-on-a-string model (65), in which G-quadruplexes are compared to beads moving independently of each other, and the stacking model (66,67), in which G-quadruplexes stack on each other in a higher-order structure. Recent molecular modeling studies suggest that the (3+1) form and parallel quadruplexes provide efficient scaffolds for a compact-stacking higher-order quadruplex structure of human telomeric DNA (68). However, as shown in the Supplementary Figure S8, the model indicates that the antiparallel chair-type G-quadruplex may provide an additional and efficient scaffold through the base-mediated stacking interaction for formation of higher-order quadruplex structure. Although further experimental data is needed to validate the model, our structure highlights the possible features required for studying the higher-order quadruplex structure of human telomeric DNA.

In summary, we revealed that *htel21*_Br-8,20 adopts a novel antiparallel chair-type topology in the K^+^ solution. Our result expands the repertoire of the polymorphic human telomeric DNA G-quadruplex structures as well as highlights the important roles of loops and water molecules in the folding topology of G-quadruplexes. Especially, the water•T5•water•T17 pseudo tetrad observed in our crystal structure of *htel21*_Br-8,20 provides a glimpse into understanding the mechanisms of the pH-dependent conformational switch of G-quadruplex. Furthermore, our coordinates are invaluable for the ligand design against G-quadruplex targets.

## DATA AVAILABILITY

Atomic coordinates and structure factors for the reported crystal structures have been deposited with the Protein Data bank under accession number 6JKN.

## Supplementary Material

gkz221_Supplemental_File
