# Supplementary material for: The crystal structure of an antiparallel chair-type G-quadruplex formed by Bromo-substituted human telomeric DNA
Source: Nucleic Acids Res. 2019 Apr 8;47(10):5395–404. doi: 10.1093/nar/gkz221 (PMC6547763; doi:10.1093/nar/gkz221)
Supplement: gkz221_Supplemental_File [file gkz221_supplemental_file.docx]

Supporting information for

**The Crystal Structure of an Antiparallel Chair-type G-quadruplex Formed by Bromo-substituted Human Telomeric DNA**

Yanyan Geng^1, †^, Changdong Liu^1, †^, Bo Zhou^1, 2, †^, Qixu Cai^1^, Haitao Miao^1^, Xiao Shi^1^ and Guang Zhu^1, 3,^ *

^1^ Division of Life Science, The Hong Kong University of Science and Technology, Clear Water Bay, Kowloon, Hong Kong SAR, China

^2^ Institute for Advanced Study, The Hong Kong University of Science and Technology, Clear Water Bay, Kowloon, Hong Kong SAR, China

^3^ State Key Laboratory of Molecular Neuroscience, The Hong Kong University of Science and Technology, Clear Water Bay, Kowloon, Hong Kong SAR, China

* To whom correspondence should be addressed. Tel: 852-2358-8705; Fax: 852-2358-1552; Email: gzhu@ust.hk

^†^ The authors wish it to be known that, in their opinion, the first 3 authors should be regarded as joint First Authors.

**Supplementary tables and figures**

**Table S1. Crystallographic data collection and refinement statistics**

| **Data collection** |  |
| --- | --- |
| Space group | P6_5_22 |
| Wavelength (Å) | 0.91904 |
| Unit cell parameters (Å) | a=b=46.252 c=120.054 α=β=90° γ=120° |
| Resolution range (Å) | 50-1.40 (1.42-1.40) |
| No. of unique reflections | 15673 (677) |
| Redundancy | 32.8 (17.2) |
| I/σ | 43.7 (1.2) |
| Completeness (%) | 99.3 (91.7) |
| R_merge_^a^ (%) | 7.5 (105.9) |
| CC_1/2_ (highest-resolution shell)^b^ | 0.869 |
| **Structure refinement** |  |
| Resolution (Å) | 38-1.40 |
| R_work_^c^ (%) | 15.90 |
| R_free_^d^ (%) | 18.42 |
| RMSD bonds (Å) | 0.009 |
| RMSD angles (°) | 1.854 |
| Average B factor (Å^2^) | 31.0 |
| No. of atoms |  |
| DNA atoms | 449 |
| Water | 119 |
| Ligands | 0 |

Numbers in parentheses represent the values for the highest-resolution shell.

^a^R_merge_ = Σ|*I*_i_ - <*I*>|/Σ*I*_i_, where *I*_i_ is the intensity of measured reflection and <*I*> is the mean intensity of all symmetry-related reflections.

^b^CC_1/2_ was defined in ([1](#_ENREF_1)).

^c^R_work_ = Σ_W_||*F*_calc_| - |*F*_obs_||/Σ|*F*_obs_|, where *F*_obs_ and *F*_calc_ are observed and calculated structure factors. W is working dataset of about 95% of the total unique reflections randomly chosen and used for refinement.

^d^R_free_ = Σ_T_||*F*_calc_| - |*F*_obs_||/Σ|*F*_obs_|, where T is a test dataset of about 5% of the total unique reflections randomly chosen and set aside prior to refinement.

**Table S2. Sugar pucker conformations of the TTA loops for: 6JKN,** *htel21*_Br-8,20 in this work; **5YEY, the human telomeric variant** *htel21***T_18_; 1KF1, the propeller-type parallel-stranded form observed for d[A(GGGTTA)_3_GGG] in a K^+^ containing crystal; 143D, the basket-type form observed for d[A(GGGTTA)_3_GGG] in Na^+^ solution; 2JSM, the (3 + 1) Form 1 observed for d[TA(GGGTTA)_3_GGG]; 2HY9, the (3 + 1) Form 1 observed for the end-modified sequence d[AAA(GGGTTA)_3_GGGAA] in K^+^ solution; 2JSL, the (3+1) Form 2 observed for d[TA(GGGTTA)_3_GGGTT]; 2JPZ, the (3+1) Form 2 observed for d[TTA(GGGTTA)_3_GGGTT] sequence in K^+^ solution; 2KF8, the basket-type form with two G-tetrad layers observed for the modified d[(GGGTTA)_3_GGGT] sequence in K^+^ solution; 2MBJ, the antiparallel (2+2) form observed for d[(TTAGGGTTA)_4_TTA] G-quadruplex in Na^+^ solution. The first structure in the NMR ensemble was used to do analysis. The numbers of the bases were aligned to 6JKN.**

|  | 6JKN | 5YEY | 1KF1 | 143D | 2JSM | 2HY9 | 2JSL | 2JPZ | 2KF8 | 2MBJ |
| --- | --- | --- | --- | --- | --- | --- | --- | --- | --- | --- |
| **T4** | C2'-*endo* | C2'-*endo* | C1'-*exo* | C2'-*endo* | C3'-*endo* | C4'-*endo* | C2'-*endo* | C2'-*endo* | C2'-*endo* | C1'-*exo* |
| **T5** | C2'-*endo* | C4'-*endo* | C3'-*endo* | C1'-*exo* | C3'-*exo* | O1'-*endo* | C2'-*endo* | O1'-*endo* | C1'-*exo* | C2'-*endo* |
| **A6** | C2'-*endo* | C2'-*endo* | C2'-*endo* | C2'-*endo* | C1'-*endo* | O1'-*endo* | C3'-*exo* | C1'-*exo* | C2'-*endo* | C2'-*endo* |
|  | | | | | | | | | | |
| **T10** | C3'-*exo* | C2'-*endo* | C1'-*exo* | C3'-*exo* | C2'-*endo* | C1'-*exo* | C4'-*exo* | C4'-*exo* | C2'-*endo* | C1'-*exo* |
| **T11** | C2'-*endo* | C2'-*exo* | C3'-*endo* | C2'-*endo* | C3'-*endo* | C1'-*exo* | C2'-*exo* | C4'-*endo* | C3'-*exo* | C1'-*exo* |
| **A12** | C2'-*endo* | C3'-*exo* | C2'-*endo* | C3'-*exo* | C2'-*endo* | O1'-*exo* | O1'-*endo* | O1'-*endo* | O1'-*endo* | C2'-*endo* |
|  | | | | | | | | | | |
| **T16** | C1'-*exo* | C2'-*exo* | C2'-*endo* | C2'-*endo* | C2'-*endo* | C2'-*endo* | C4'-*exo* | C2'-*exo* | C3'-*exo* | C2'-*endo* |
| **T17** | C2'-*endo* | C3'-*exo* | C3'-*endo* | C3'-*exo* | C2'-*endo* | C3'-*endo* | C3'-*endo* | C2'-*endo* | C1'-*exo* | C2'-*endo* |
| **A18** | C2'-*endo* |  | C2'-*endo* | C2'-*endo* | C2'-*endo* | C4'-*exo* | C4'-*exo* | C4'-*endo* | O1'-*endo* | C3'-*exo* |

**Figure S1 Schematic topology representation of eight structures of intramolecular G-quadruplexes formed by four-repeat human telomeric sequences in the present study: (A) The basket-type form observed for d[A(GGGTTA)_3_GGG] in Na^+^ solution, (B) the propeller-type parallel-stranded form observed for d[A(GGGTTA)_3_GGG] in a K^+^ containing crystal, (C) the (3 + 1) Form 1 observed for d[TA(GGGTTA)_3_GGG] and the end-modified sequence d[AAA(GGGTTA)_3_GGGAA] in K^+^ solution, (D) the (3+1) Form 2 observed for d[TA(GGGTTA)_3_GGGTT] and d[TTA(GGGTTA)_3_GGGTT] sequence in K^+^ solution, (E) the basket-type form with two G-tetrad layers observed for the modified d[(GGGTTA)_3_GGGT] sequence in K^+^ solution, and (F) the antiparallel (2+2) form observed for d[(TTAGGGTTA)_4_TTA] G-quadruplex in Na^+^ solution. *anti* guanines are colored in cyan, while *syn* guanines are colored in magenta.**

**
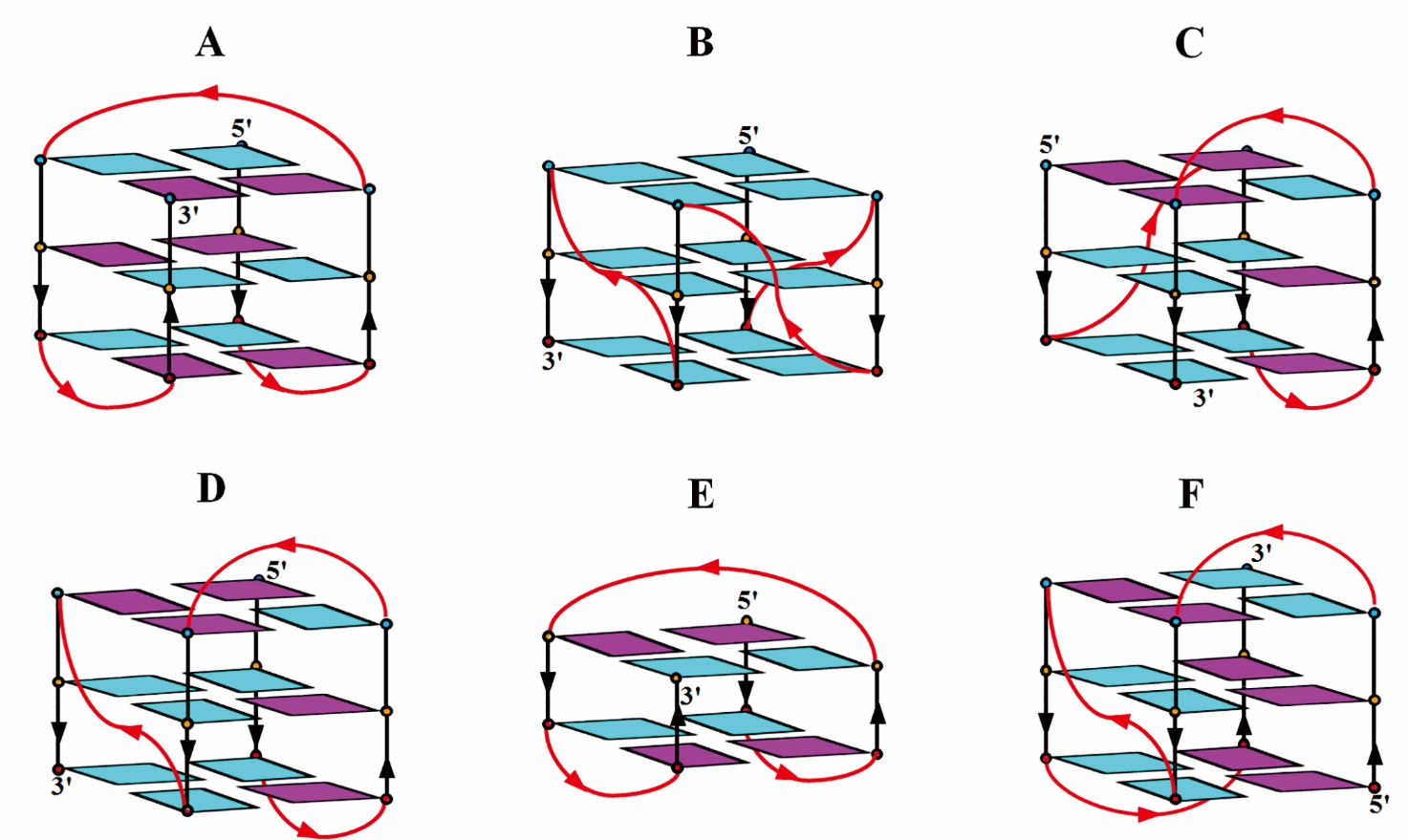
**

**Figure S2 (A) The 8-bromoguanine** (^8Br^G_8_) **substitutions at various G positions of** *hetl21*. (B) CD spectra and (C) imino regions of 1D ^1^H NMR spectra for the sequeces shown in (A).

A.

| Name | Sequence | | | | | | |
| --- | --- | --- | --- | --- | --- | --- | --- |
| *htel21* | GGG | TTA | GGG | TTA | GGG | TTA | GGG |
| *htel21*_Br-8 | GGG | TTA | G(^8Br^G_8_)G | TTA | GGG | TTA | GGG |
| *htel21*_Br-20 | GGG | TTA | GGG | TTA | GGG | TTA | G(^8Br^G_20_)G |
| *htel21*_Br-1,13 | (^8Br^G_1_)GG | TTA | GGG | TTA | (^8Br^G_13_)GG | TTA | GGG |
| *htel21*_Br-8,20 | GGG | TTA | G(^8Br^G_8_)G | TTA | GGG | TTA | G(^8Br^G_20_)G |
| *htel21*_Br-1,8,13 | (^8Br^G_1_)GG | TTA | G(^8Br^G_8_)G | TTA | (^8Br^G_13_)GG | TTA | GGG |
| *htel21*_Br-1,7,8,13,19,20 | (^8Br^G_1_)GG | TTA | (^8Br^G_7_^8Br^G_8_)G | TTA | (^8Br^G_13_)GG | TTA | (^8Br^G_19_^8Br^G_20_)G |

B.


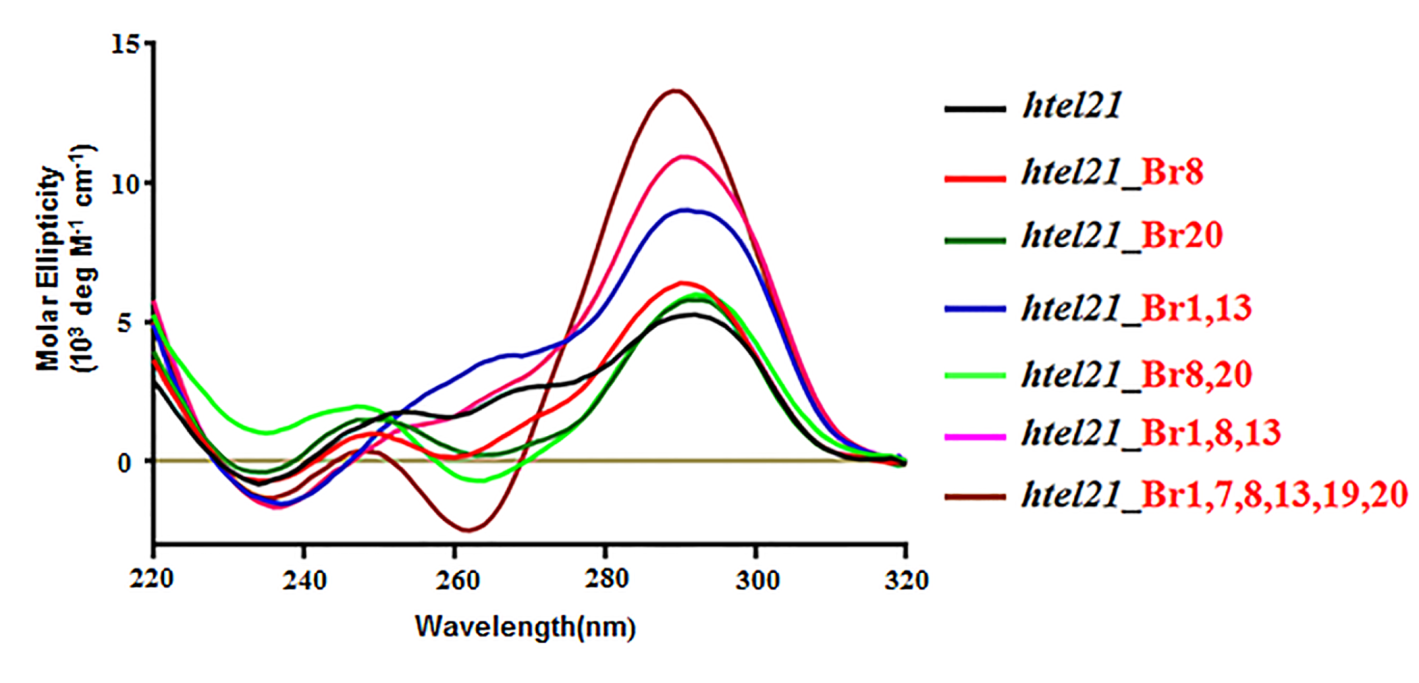


C.


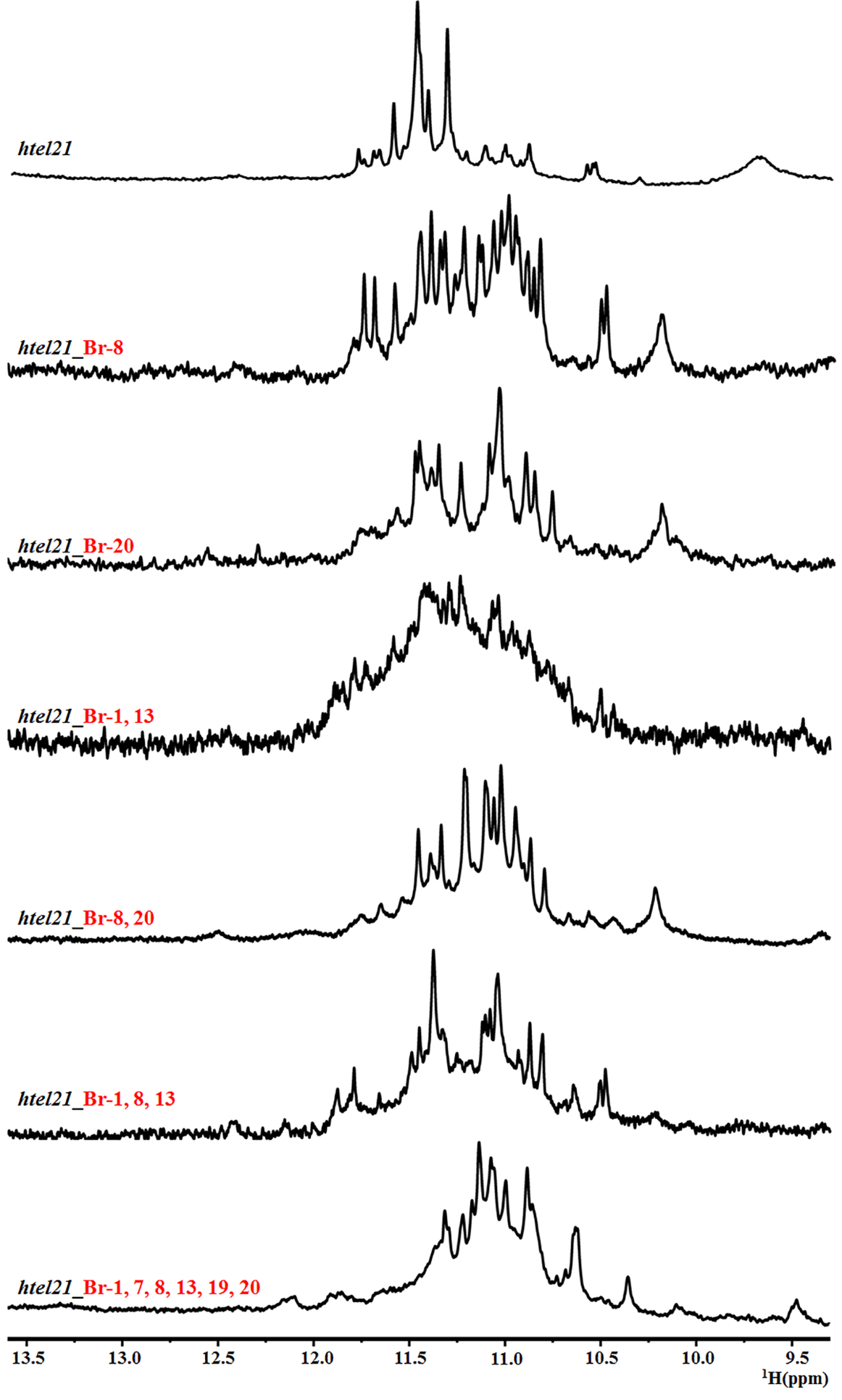


**Figure S3**. (A) Names and sequences used in this study and CD melting temperature (T_m_) of G-quadruplexes in 70 mM K^+^ solution. (B) CD spectra of *htel21*, *htel21*_Br-8, *htel21*_Br-20 and *htel21*_Br-8,20 in 70 mM KCl, 20 mM potassium phosphate solution recorded at 25°C. (C) CD melting experiments of *htel21*, *htel21*_Br-8, *htel21*_Br-20 and *htel21*_Br-8,20.

**A**

| Name | Sequence | | | | | | | T_m_(℃) |
| --- | --- | --- | --- | --- | --- | --- | --- | --- |
| *htel21* | GGG | TTA | GGG | TTA | GGG | TTA | GGG | 74 |
| *htel21*_Br-8 | GGG | TTA | G(^8Br^G_8_)G | TTA | GGG | TTA | GGG | 77 |
| *htel21*_Br-20 | GGG | TTA | GGG | TTA | GGG | TTA | G(^8Br^G_20_)G | 78 |
| *htel21*_Br-8,20 | GGG | TTA | G(^8Br^G_8_)G | TTA | GGG | TTA | G(^8Br^G_20_)G | 77 |

**
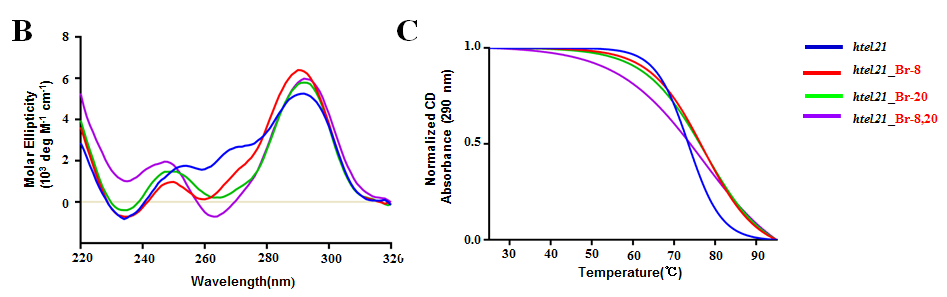
**

**Figure S4**. (A) Top: The H8 proton of guanine at positon 20 in *htel21*_Br-8 was assigned with 2D ^13^C-^1^H HSQC spectra of samples containing site-specific low-enrichment (5%) ^15^N, ^13^C^13^-labelled oligonucleotides at the position 20; Bottom: Expanded ^1^H-^1^H NOESY spectrum (50 ms mixing time) correlating base H8 and sugar H1' protons of *htel21*_Br-8. The intraresidue guanosine H8-H1' cross-peak indicates *syn* glycosidic bond. (B) Top: The H8 proton of guanine at positon 8 in *htel21*_Br-20 was assigned with 2D ^13^C-^1^H HSQC spectra of samples containing site-specific low-enrichment (5%) ^15^N, ^13^C-labelled oligonucleotides at the position 8; Bottom: Expanded ^1^H-^1^H NOESY spectrum (50 ms mixing time) correlating base H8 and sugar H1' protons of *htel21*_Br-20. The intraresidue guanosine H8-H1' cross-peak indicates *syn* glycosidic bond. Spectra were recorded at 800 MHz at 25 °C in 100% D_2_O, 70 mM KCl, 20 mM potassium phosphate buffer (pH 7.0)

**
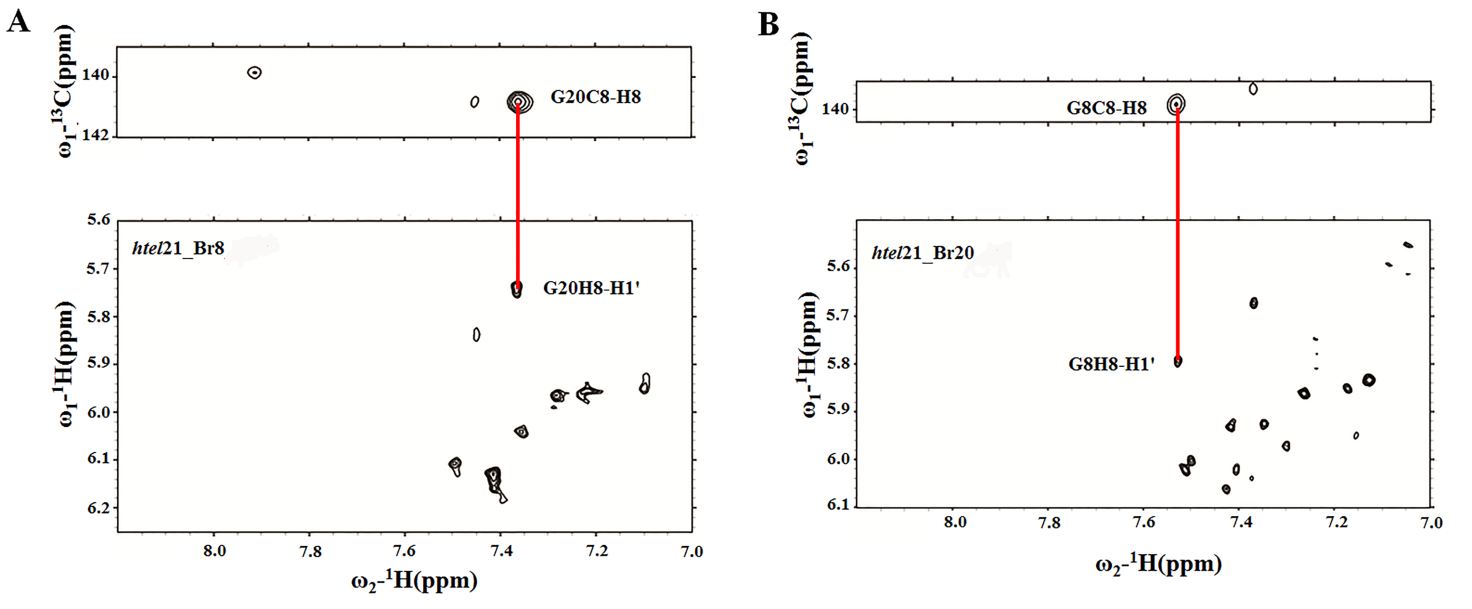
**

**Figure S5**. Representative portion of the 2Fo-Fc electron density map, contoured at 1.0 σ. The *htel21*_Br-8,20 is shown in sticks, water molecules as red spheres, and potassium ions as purple spheres.


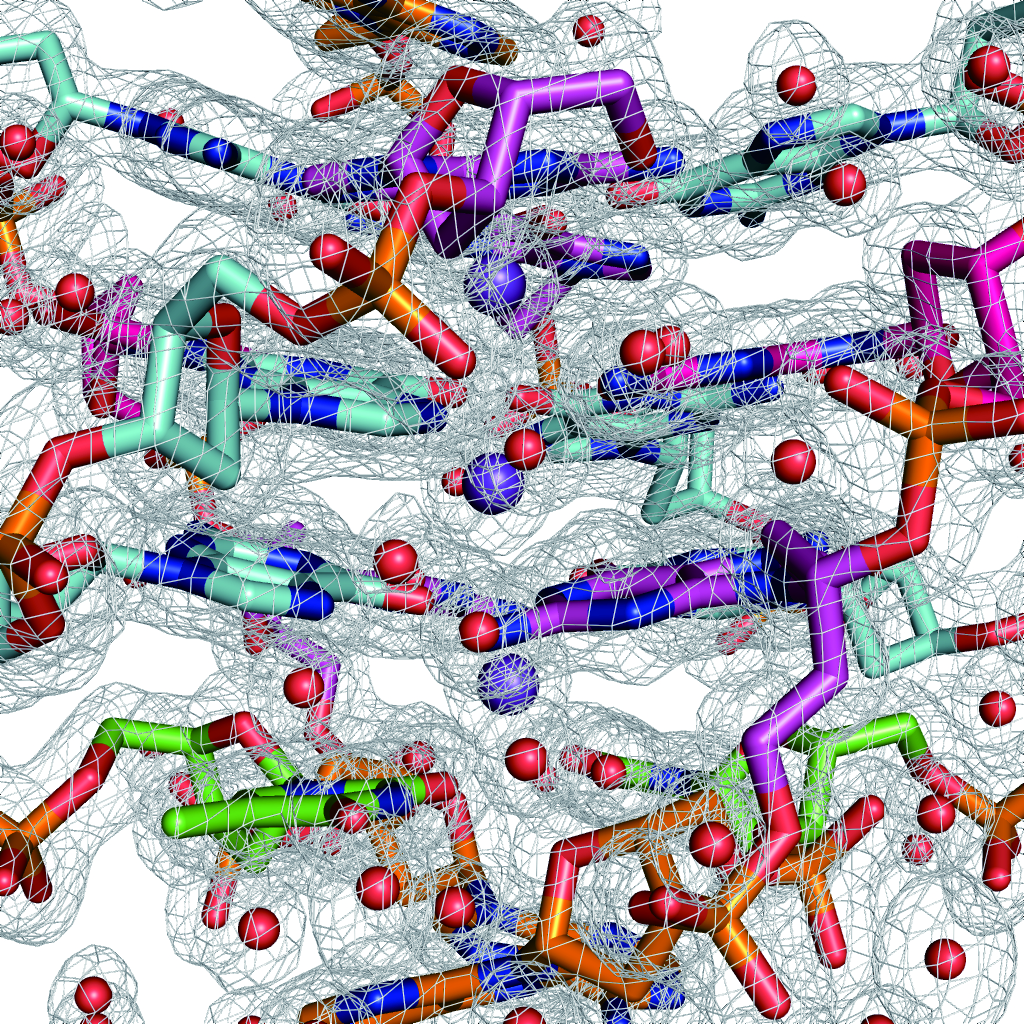


**Figure S6**. Intermolecular π−π packing interactions and a canonical Watson-Crick A12•T11' base pair with hydrogen bonds shown as dashed red lines in *htel21*_Br-8,20 crystal. The prime (′) notation signifies that the two bases belong to separate oligonucleotide strands.


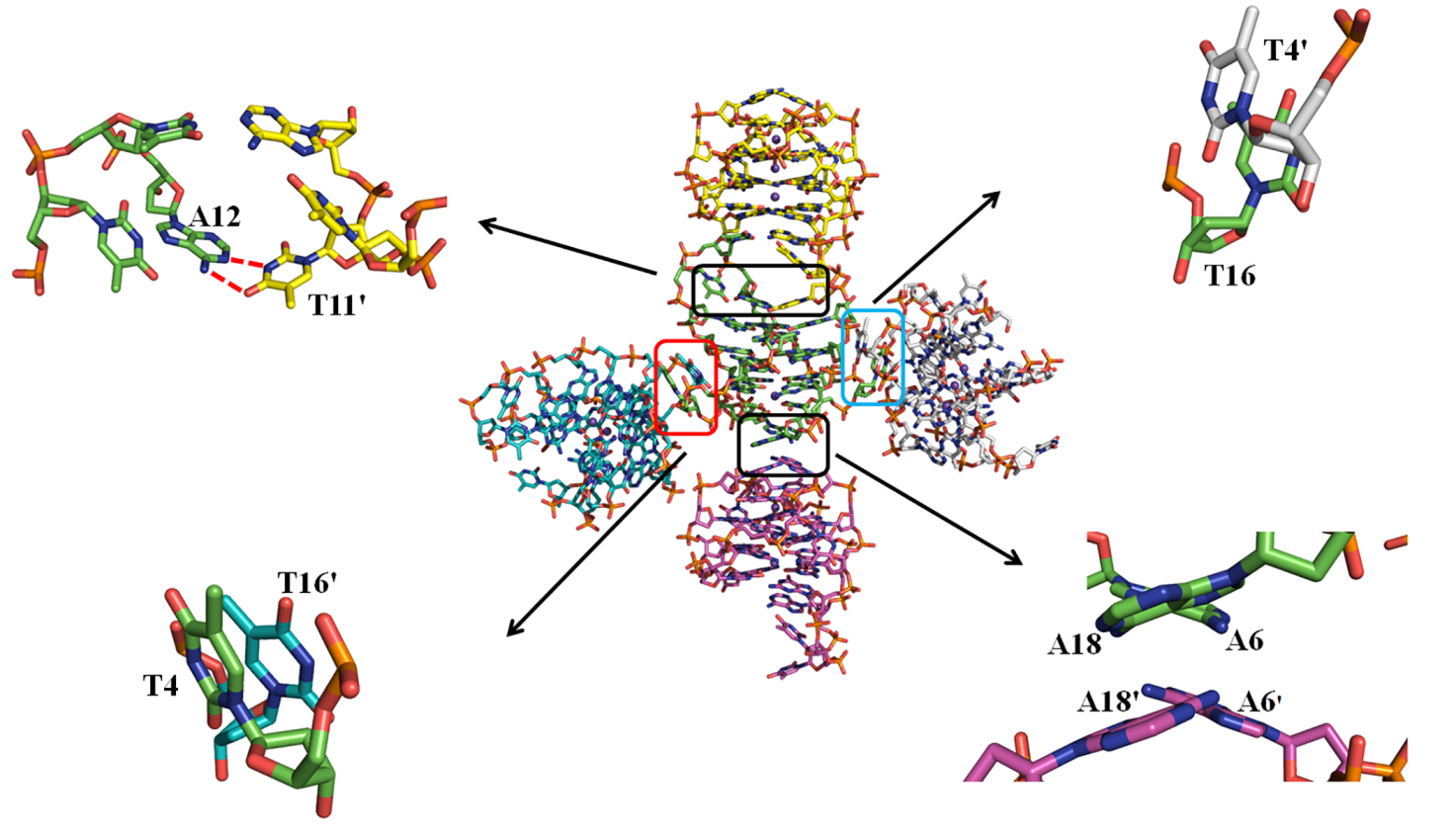


**Figure S7**. (A) The distribution of dihedral angles in *htel21*_Br-8,20 (PDB code :6JKN). (B), (C) and (D) Comparison of torsion angles of TTA loops with the structures shown in Table S2 and the Supplementary Figure S1. Six dihedral angles, α, β, γ, δ, ε and ζ describe DNA backbone ; dihedral angle χ refers to *syn* vs *anti* nucleobase conformation. **The numbers of the bases were aligned to** *htel21*_Br-8,20**. The close conformation of (E) the third loop in 2MBJ and (F) the second loop in 2JSL (colored by cyan) compared with the T4-T5-A6 and T10-T11-A12 in** *htel21*_Br-8,20 **(colored by magenta).**


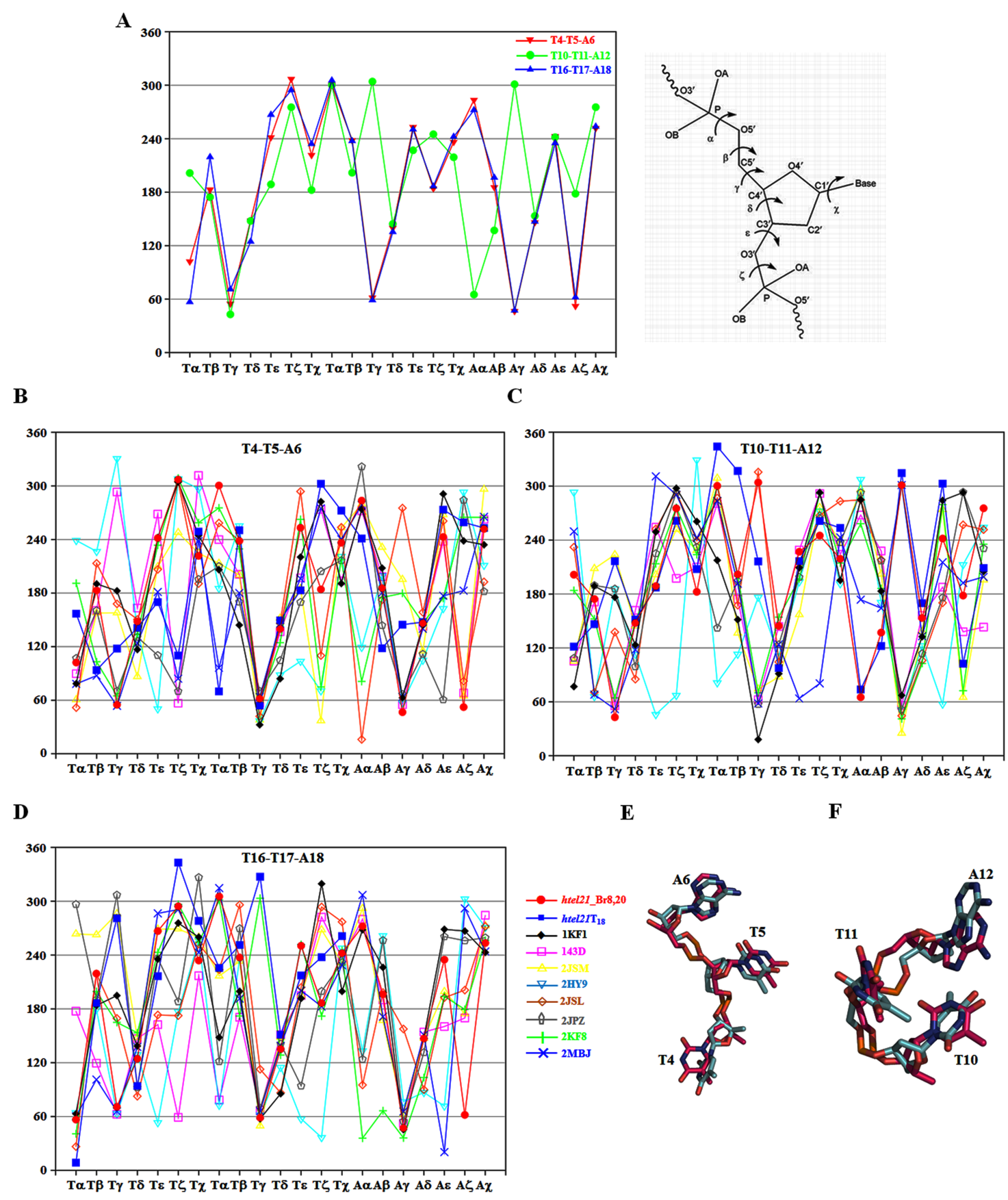


**Figure S8**. The model for higher-order quaruplex structrures formed by long human telomeric DNA sequences through antiparallel G-quadurplex unit**.**


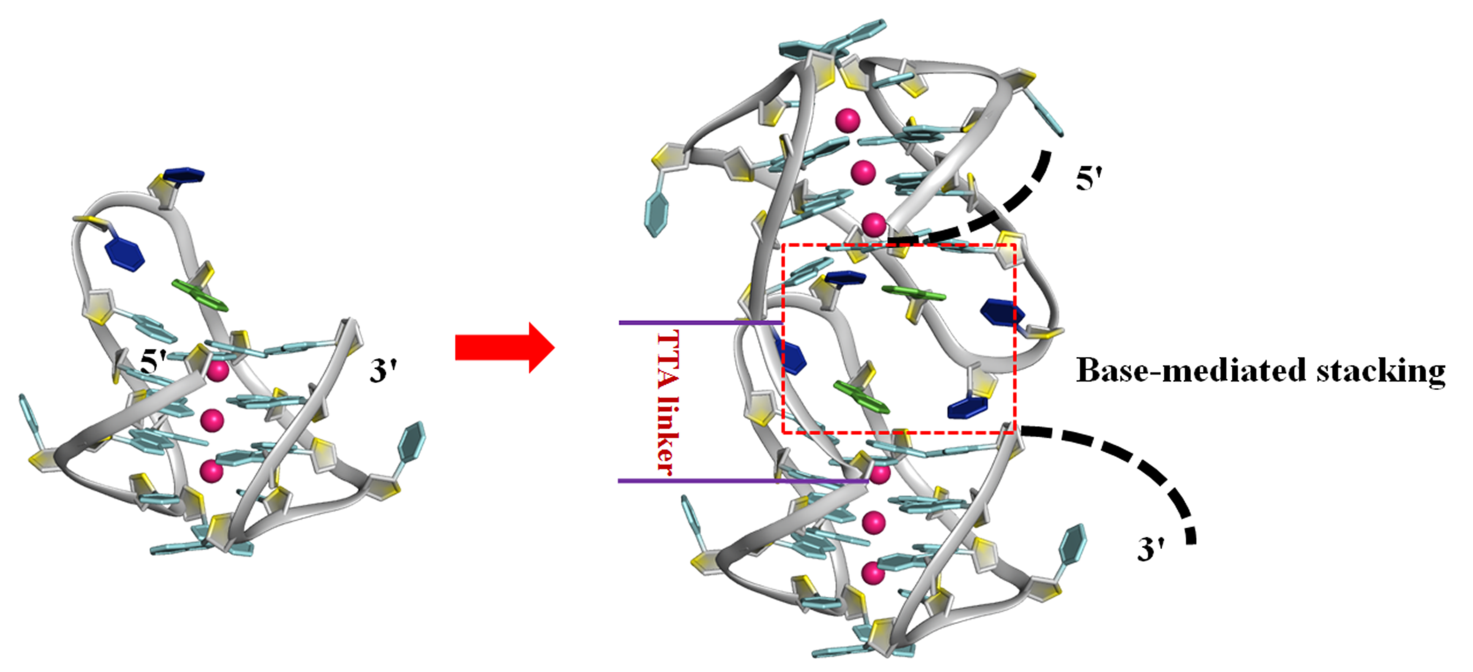


**Supplementary References**

1. Karplus, P.A. and Diederichs, K. (2012) Linking crystallographic model and data quality. *Science*, **336**, 1030-1033.
